# Mitochondria-targeted antioxidant supplementation improves 8 km time trial performance in middle-aged trained male cyclists

**DOI:** 10.1186/s12970-021-00454-0

**Published:** 2021-08-21

**Authors:** S. C. Broome, A. J. Braakhuis, C. J. Mitchell, T. L. Merry

**Affiliations:** 1 Discipline of Nutrition, School of Medical Sciences, University of Auckland, Private Bag 92019, Auckland, New Zealand; 2 School of Kinesiology, University of British Columbia, Vancouver, Canada; 3 Maurice Wilkins Centre for Molecular Biodiscovery, University of Auckland, Auckland, New Zealand

**Keywords:** ROS, Mitochondria, Antioxidant, Performance, Oxidative stress

## Abstract

**Background:**

Exercise increases skeletal muscle reactive oxygen species (ROS) production, which may contribute to the onset of muscular fatigue and impair athletic performance. Mitochondria-targeted antioxidants such as MitoQ, which contains a ubiquinone moiety and is targeted to mitochondria through the addition of a lipophilic triphenylphosphonium cation, are becoming popular amongst active individuals as they are designed to accumulate within mitochondria and may provide targeted protection against exercise-induced oxidative stress. However, the effect of MitoQ supplementation on cycling performance is currently unknown. Here, we investigate whether MitoQ supplementation can improve cycling performance measured as time to complete an 8 km time trial**.**

**Method:**

In a randomized, double-blind, placebo-controlled crossover study, 19 middle-aged (age: 44 ± 4 years) recreationally trained (VO_2peak_: 58.5 ± 6.2 ml·kg^− 1^·min^− 1^, distance cycled per week during 6 months prior to study enrollment: 158.3 ± 58.4 km) male cyclists completed 45 min cycling at 70% VO_2peak_ followed by an 8 km time trial after 28 days of supplementation with MitoQ (20 mg·day^− 1^) and a placebo. Free F_2_-isoprostanes were measured in plasma samples collected at rest, after 45 min cycling at 70% VO_2peak_ and after completion of the time trial. Respiratory gases and measures of rating of perceived exertion (RPE) were also collected.

**Results:**

Mean completion time for the time trial was 1.3% faster with MitoQ (12.91 ± 0.94 min) compared to placebo (13.09 ± 0.95 min, *p* = 0.04, 95% CI [0.05, 2.64], *d* = 0.2). There was no difference in RPE during the time trial between conditions (*p* = 0.82) despite there being a 4.4% increase in average power output during the time trial following MitoQ supplementation compared to placebo (placebo; 270 ± 51 W, MitoQ; 280 ± 53 W, *p* = 0.04, 95% CI [0.49, 8.22], *d* = 0.2). Plasma F_2_-isoprostanes were lower on completion of the time trial following MitoQ supplementation (35.89 ± 13.6 pg·ml^− 1^) compared to placebo (44.7 ± 16.9 pg·ml^− 1^ *p* = 0.03).

**Conclusion:**

These data suggest that MitoQ supplementation may be an effective nutritional strategy to attenuate exercise-induced increases in oxidative damage to lipids and improve cycling performance.

## Introduction

Oxygen-derived radical and non-radical species, collectively referred to as reactive oxygen species (ROS), are continuously produced by skeletal muscle [1]. High levels of ROS production in cells may overwhelm the endogenous antioxidant defense network resulting in damage to cellular proteins, lipids and DNA and impaired cellular function. Chronic oxidative stress is deleterious to muscle function [2] and has been associated with the pathogenesis of several diseases, including cardiovascular disease [3] and type 2 diabetes [4]. Interestingly, while regular exercise is known to promote a wide range of health benefits, it also results in an acute increase in ROS levels systemically and in skeletal muscle [1].

Despite the traditional view that ROS are primarily harmful molecules, more recent evidence has shown that transient and physiological exercise-induced increases in skeletal muscle ROS production are required for optimal contractile function [5, 6]. Furthermore, some studies have shown that antioxidant supplementation can attenuate exercise-induced redox signalling and training adaptations [7–9], which may translate to impaired training-induced improvements in performance [10–12]. However, while exercise-induced ROS play an important role in adaptation, acute exposure to O_2_·^−^ and H_2_O_2_ can impair sarcoplasmic reticulum calcium release and myofibrillar calcium sensitivity resulting in the development of muscular fatigue [13–16]. Thus, increasing the capacity of skeletal muscle to neutralise ROS by using exogenous antioxidant supplements has received much attention as a potential strategy to delay the onset of muscular fatigue and improve athletic performance. Several general antioxidants such as N-acetylcysteine and vitamin C and E have been studied for their effectiveness as ergogenic aids. While the results have been mixed, if not mostly disappointing [17, 18], there is some evidence to support the use of antioxidant supplements to improve performance. Vitamin C and N-acetylcysteine have been shown to improve performance when they are used to reverse a particular antioxidant deficiency [19]. Furthermore, N-acetylcysteine appears to inhibit muscular fatigue when infused intravenously during [15, 16] or immediately before exercise [20], and acute oral supplementation with N-acetylcysteine has been shown to improve performance during repetitive handgrip exercise [21] and repeated bouts of running [22].

Multiple enzymes and organelles within skeletal muscle generate ROS during exercise. NADPH oxidase enzymes are thought to be the primary source of exercise-induced O_2_·^−^/H_2_O_2_ since the increase in ATP demand during exercise decreases O_2_·^−^ production by mitochondria [18, 23]. While total mitochondrial O_2_·^−^ production decreases during exercise, mitochondria continue to produce O_2_·^−^ at complex I under conditions mimicking aerobic exercise [23] and this may contribute to the development of fatigue. Coenzyme Q10 supplementation has gained considerable attention as a dietary strategy with the potential to improve athletic performance as coenzyme Q10 plays an essential role in mitochondrial bioenergetics and acts as an antioxidant within plasma membranes [24]. However, accumulation of coenzyme Q10 within mitochondria is limited due to its high molecular weight and low aqueous solubility, which may explain why studies investigating the ergogenic effects of coenzyme Q10 supplementation have produced mixed results [18].

Mitochondria-targeted coenzyme Q10, known as Mitoquinone (MitoQ), consists of a ubiquinone moiety conjugated to a lipophilic triphenylphosphonium cation. The triphenylphosphonium cation facilitates the accumulation of MitoQ within mitochondria 100–1000-fold compared to that found in the cytoplasm, a process that is driven by the plasma and mitochondrial membrane potentials [25, 26]. Within mitochondria, MitoQ is reduced by complex II to ubiquinol, which prevents lipid peroxidation directly by acting as a chain-breaking antioxidant and indirectly by recycling the α-tocopheroxyl radical to its active form [27, 28]. Williamson et al. [29] recently showed that MitoQ protects against exercise-induced increases in mitochondrial DNA damage; however, the effects of MitoQ on athletic performance are unknown. The aim of this study was to investigate whether MitoQ supplementation could improve the time taken to complete an 8 km cycling time trial, which was selected because antioxidant treatment has previously been shown to improve performance during very high intensity, short duration cycling [15]. Therefore, it was hypothesized that MitoQ would improve 8 km cycling time trial performance compared to placebo.

## Methods

### Participants

**Fig. 1:**
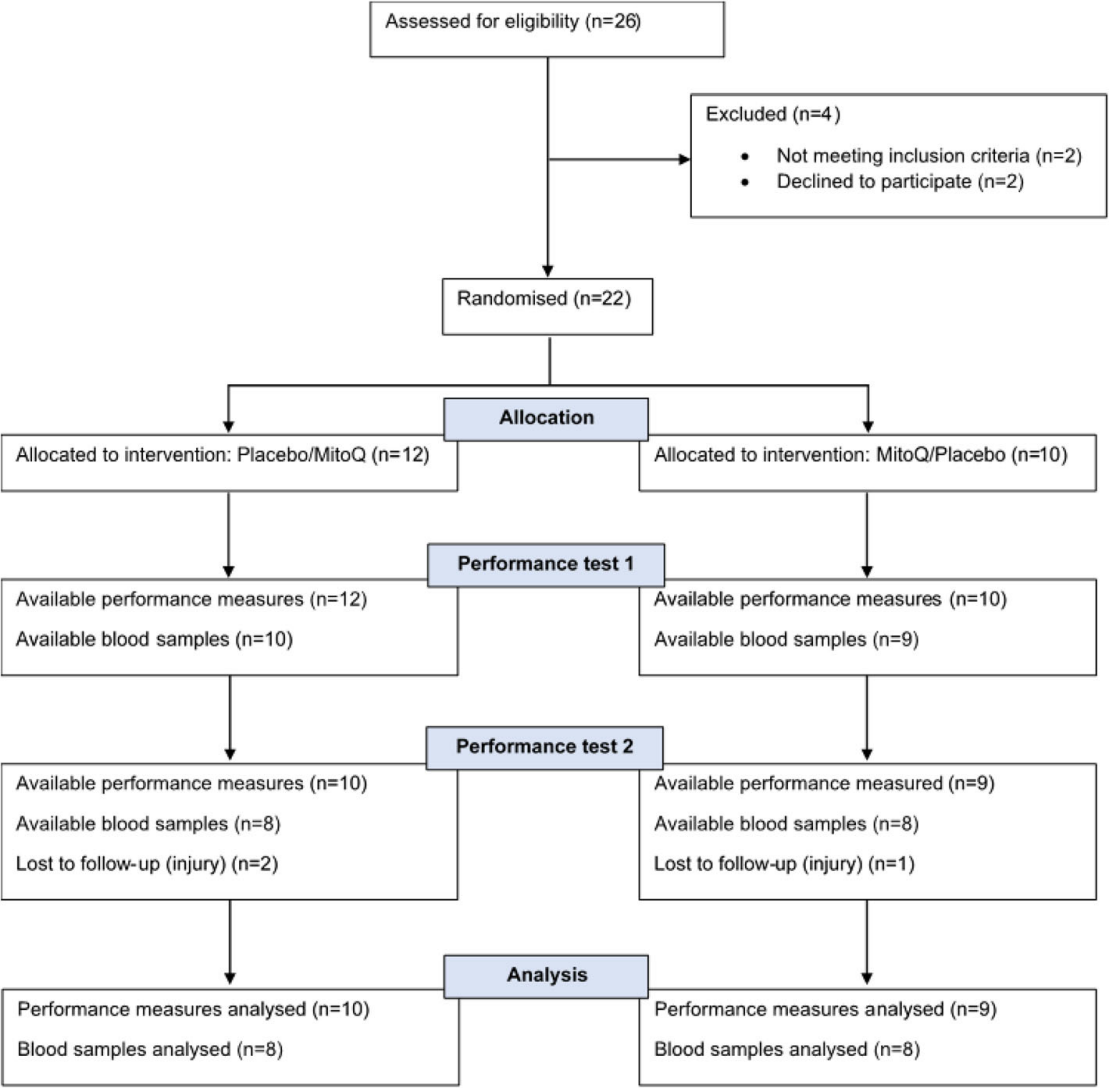
CONSORT flow chart. This figure shows the flow of patients through the trial according to the criteria recommended in the CONSORT Guidelines

Twenty-two healthy middle-aged, recreationally trained male cyclists were recruited via advertisements. Three participants withdrew from the study due to injury (Fig. 1), and results are presented for the 19 participants who completed all study visits. Participant characteristics are given in Table 1, and training records indicated that participants cycled 158.3 ± 58.4 km per week on average during the 6 months prior to their enrollment in the study. Participants were non-smokers, free of injury and chronic illnesses including cardiovascular and metabolic disease, not taking medication that may influence cycling performance and had not taken antioxidant supplements within 8 weeks of study enrollment. All participants provided written informed consent before the commencement of the study. The study was approved by the Northern Health and Disability Ethics Committee (New Zealand) (18/CEN/136) on 3rd September 2018, registered with the Australia New Zealand Clinical Trial Registry (ACTRN12619000451101) on 19th March 2019 and conducted in accordance with the ethical standards laid down in the Declaration of Helsinki.

**Table 1:** Participant characteristics

Characteristics	Mean (***n*** = 19)
Age (years)	44 ± 4
Height (cm)	178.2 ± 4.8
Weight (kg)	78.5 ± 10.2
BMI (kg/m^2^)	24.7 ± 2.4
VO_2peak_ (ml·kg^−1^·min^− 1^)	58.5 ± 6.2
Power output at VO_2peak_ (W)	398 ± 40
Power output eliciting 70% VO_2peak_ (W)	224 ± 28

Values are mean ± SD

### Experimental design

**Fig. 2:**
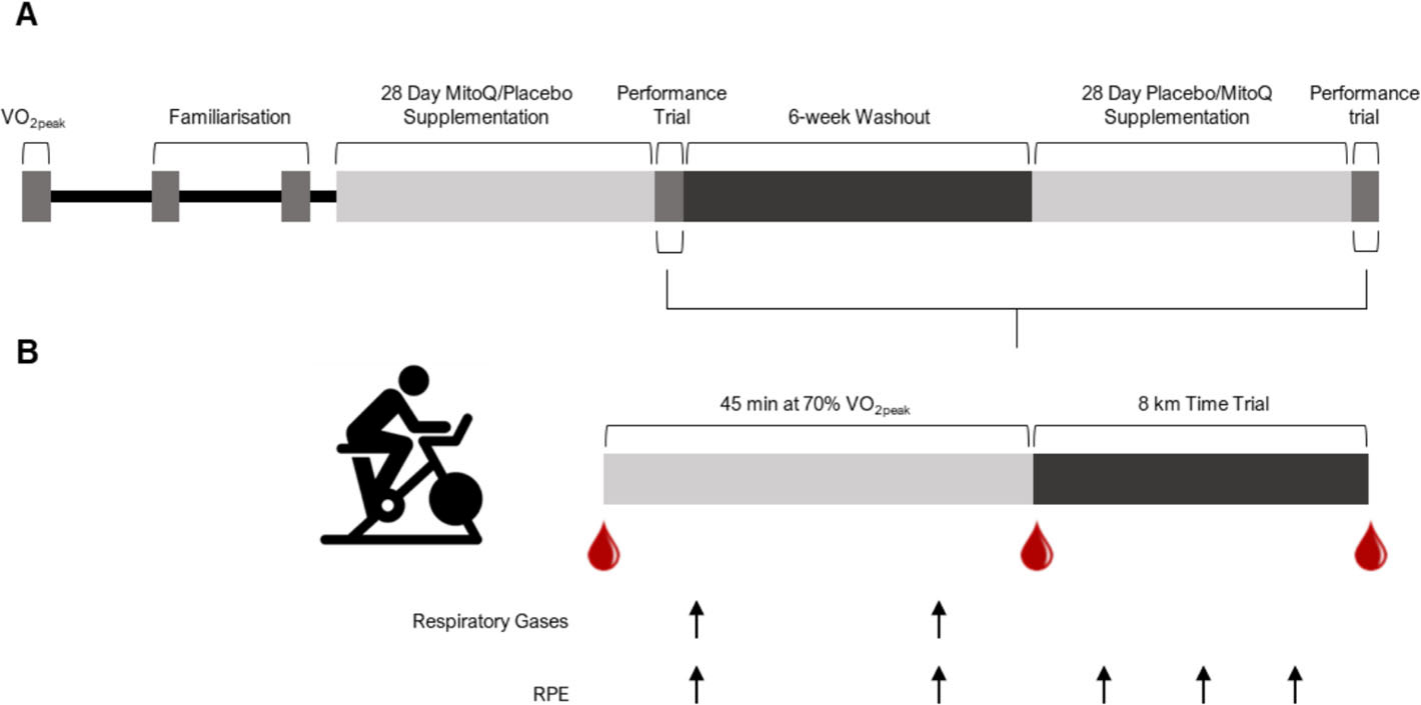
Study timeline (**A**) and performance trial procedures (**B**)

The study was a double-blind, placebo-controlled crossover design (Fig. 2). Participants attended the laboratory at The University of Auckland, New Zealand on five occasions over 15 weeks between March and December 2019 to complete an incremental test to exhaustion (VO_2peak_), two familiarisation rides and two performance trials on a stationary cycle ergometer (Velotron, RacerMate, Seattle, WA). The performance trial consisted of 45 min cycling at a fixed workload eliciting 70% VO_2peak_ followed by an 8 km time trial. All tests were performed under normal laboratory conditions (temperature: 22.18 ± 1.24 °C, relative humidity: 39.82 ± 6.18%) and were supervised by the same researcher who was blinded to the experimental condition. Consistent encouragement was provided across time trials in the form of predetermined verbal phrases every 0.5 km.

### Incremental test to exhaustion (VO_2peak_)

On the first laboratory visit, participants completed an incremental ramped exercise test on a cycle ergometer to determine VO_2peak_. The handlebars and seat were positioned to match that of the participant’s own bike, and this setup was the same for all subsequent trials. The test protocol started at 125 W and increased at a rate of 25 W·min^− 1^ until voluntary exhaustion. Participants pedalled at a self-selected cadence between 70 and 90 rpm, and the test ended when cadence dropped below 70 rpm for more than 10 s. VO_2peak_ was determined as the highest 30-s average value attained using a metabolic system (Parvo Medics True One 2400, Sandy, UT) and workload corresponding to VO_2peak_ was recorded.

### Supplementation

In a double blind, placebo-controlled design, participants were randomized by an independent researcher to two groups, which would determine the order in which they received MitoQ (mitoquinone mesylate 20 mg·day^− 1^, Alaron; Nelson, New Zealand) and an identical placebo (Alaron [tapioca powder, precipitated silica and microcrystalline cellulose 101]). Order and sequence effects were controlled for through counterbalancing. Tablets were dispensed into unmarked bottles by the independent researcher who performed the randomisation, and these bottles were given to the researcher conducting the trial for distribution to participants. The researcher conducting the trial remained blinded throughout data collection. Participants were instructed to consume one tablet per day orally 30 min before breakfast for 28 days before completing the first performance trial. The selection of a 28-day supplementation period was informed by previous human research [30]. After a 6-week washout period, participants crossed over into the other treatment group before completing the second performance trial. Supplementation adherence was monitored by oversupplying the participants with tablets and counting the number of tablets returned at each performance trial.

### Performance trial

Participants arrived at the laboratory at 8 am having abstained from alcohol consumption and exercise for the preceding 48 and 24 h, respectively. Participants rested in a supine position for 15 min before the collection of a resting blood sample. A standardized warm-up was completed, which involved cycling for 5 min at 100 W, followed by 1 min at 50, 60, 70, 80 and 90% peak power output (determined during the VO_2peak_ test), then 5 min at 100 W. Participants then cycled at a workload that elicited 70% VO_2peak_ for 45 min. Rating of perceived exertion (RPE) was measured using the Borg scale (6–20), and respiratory gases were collected at 15- and 30-min for 3 min. Participants then immediately completed an 8 km time trial, which they were instructed to finish in the shortest time possible. The time trial mode of the cycle ergometer allowed for the use of self-selected gearing. Visual feedback, including distance and time elapsed, speed, power and cadence was provided for the first 0.8 km of the time trial, after which the only feedback visible was the distance completed. During the time trial, RPE was measured at 2, 4 and 6 km. Blood samples were collected immediately after completion of the 45 min cycling at 70% VO_2peak_ and the time trial (Fig. 1b).

### Blood sample collection and analysis

On arrival at the laboratory, a cannula was inserted into an antecubital forearm vein. At each blood sampling time point, 10 ml of blood was collected into a vacutainer tube containing EDTA, whilest 5 ml of blood was collected into a tube containing EDTA, butylated hydroxytoluene and reduced glutathione for later analysis of plasma F_2_-isoprostanes. Blood samples were centrifuged at 2000 g for 10 min at  4°C and plasma was recovered and stored at − 80°C for later analysis. Plasma lactate, glucose, non-esterified fatty acid (NEFA) and triglyceride concentration were measured using a Roche C311 autoanalyser (Roche, Mannheim, Germany) by enzymatic colorimetric assay. Free F_2_-isoprostanes were measured in plasma using a commercial ELISA kit (Cayman Chemical, Ann Arbor, MI, USA) according to the manufacturer’s instructions.

### Dietary intake

Participants were instructed to consume their habitual diet and record their diet in a food diary for 3 days before the performance trials. The participant’s food diary for the first performance trial was copied and returned to them so that they could replicate this diet for the second, crossover performance trial. Standard evening and breakfast meals were provided for consumption on the night before and morning of each performance trial. The evening meal consisted of a chicken and pesto pasta (548 kcal, 75 g carbohydrate, 43 g protein, 9 g fat, Muscle Chow; Auckland, New Zealand), while the breakfast meal consisted of two 86 g One Square Meal bars (694 kcal, 90.4 g carbohydrate, 16.7 g protein, 23.3 g fat, Cookie Time Limited; Christchurch, New Zealand). Participants were instructed to consume the breakfast meal 90 min before the start of the performance trial.

### Habitual training load

Participants were asked to maintain a consistent and habitual training load throughout the study and to prepare for each performance trial as if it was a competitive event. The duration and mean power output of all training sessions were recorded using the exercise tracking application Strava and the participant’s own devices.

### Statistical analyses

Statistical analyses were performed using Prism 8 (GraphPad Software, Version 8), with statistical significance determined as *p* ≤ 0.05. Data are presented as means ± SD unless otherwise specified. Prior to analysis, data were assessed for normality using the D’Agostino & Pearson test, and all followed a Gaussian distribution. A paired-samples t-test was used to assess the difference in time taken to complete the time trial following MitoQ and placebo supplementation, which was the primary outcome measure in this study. Paired-samples t-tests were also used to analyse differences in power output during the time trial, heart rate during cycling at 70% VO_2peak_, and average daily training duration, mean power output during each training session and adherence to the supplementation protocol during MitoQ and placebo supplementation phases. Two-way (time x treatment) repeated measures ANOVA with Holm-Sidak’s post hoc analysis were used to analyse VO_2_, RER, RPE, and carbohydrate and fat oxidation rates during cycling at 70% VO_2peak_. Power output during each km of the time trial, RPE during the time trial and plasma glucose, NEFA, triglyceride, lactate and F_2_-isoprostane concentration were also analysed using two-way (time x treatment) repeated measures ANOVA with Holm-Sidak’s post hoc analysis. The relationship between resting F_2_-isoprostanes and exercise-induced changes in F_2_-isoprostanes and change in time to complete the time trial following MitoQ supplementation compared to placebo were analysed using linear regression. Linear regression was also used to analyse the association between power output during the time trial and plasma lactate concentration on completion of the time trial following MitoQ and placebo supplementation. Effect sizes were calculated using Cohen’s *d* and interpreted using the following quantitative statements, > 0.2 small, > 0.5 medium and > 0.8 large. Uncertainty in the estimate of the effect on time trial performance and plasma F_2_-isoprostanes is expressed as 95% confidence intervals (CI).

## Results

Supplementation adherence was > 90% and there was no difference in supplementation adherence during MitoQ and placebo supplementation periods (placebo; 94 ± 10%, MitoQ; 95 ± 6% *p* = 0.80). No side effects of supplementation were reported. A similar average daily training duration (placebo; 41.56 ± 20.12 min, MitoQ; 46.33 ± 21.15 min, *p* = 0.17) and mean power output during each training session (placebo; 169 ± 56 W, MitoQ; 172 ± 43 W, *p* = 0.75) were recorded during the placebo and MitoQ supplementation phases. Furthermore, there was no difference in nutritional intake during the 3 days preceding MitoQ and placebo supplemented trials (total energy intake, placebo; 6449 ± 2108 kcal, MitoQ; 7176 ± 2825 kcal, *p* = 0.17, carbohydrate intake, placebo; 550 ± 270 g, MitoQ; 679 ± 492 g, *p* = 0.24, fat intake, placebo; 313 ± 123 g, MitoQ; 316 ± 158 g, *p* = 0.90, protein intake, placebo; 344 ± 114 g, MitoQ; 353 ± 112 g, *p* = 0.63).

### Fixed-load cycling

During cycling at 70% VO_2peak_, a similar VO_2_, RER, carbohydrate and fat oxidation and RPE were observed following MitoQ and placebo supplementation (Table 2). Heart rate during cycling at 70% VO_2peak_ was not different between supplementation conditions (placebo; 151 ± 16 bpm, MitoQ; 153 ± 13 bpm, *p* = 0.34).

**Table 2:** Physiological response to cycling at a fixed load. VO_2_, RER, carbohydrate and fat oxidation rates and RPE at 15 and 30 min during 45 min cycling at 70% VO_2peak_

	Placebo		MitoQ	
	15 min	30 min	15 min	30 min
VO_2_ (ml·kg^− 1^·min^− 1^)	40.6 ± 4.1	41.8 ± 4.4	40.5 ± 5.2	41.4 ± 5.2
RER	0.89 ± 0.04	0.90 ± 0.04	0.89 ± 0.03	0.89 ± 0.03
Carbohydrate oxidation (g·min^− 1^)	2.84 ± 0.84	2.98 ± 0.82	2.74 ± 0.59	2.87 ± 0.60
Fat oxidation (g·min^− 1^)	0.55 ± 0.23	0.54 ± 0.23	0.58 ± 0.17	0.56 ± 0.14
RPE	13.1 ± 1.3	13.8 ± 1.7	12.8 ± 1.6	13.7 ± 1.8

Values are mean ± SD; RER, respiratory exchange ratio; RPE, rating perceived exertion

### 8 km cycling time trial performance

**Fig. 3:**
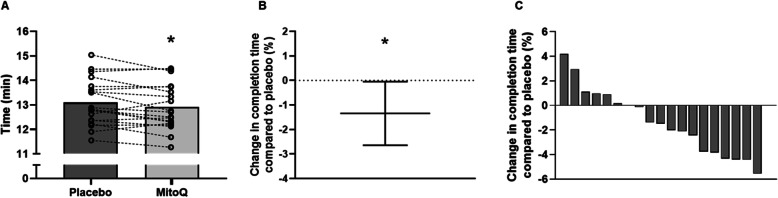
Mean time to complete the time trial following 6 weeks of placebo and MitoQ supplementation (**A**), percent change in time to complete time trial with MitoQ compared to placebo, data are presented as mean ± 95% CI (**B**), and individual percent change in time to complete the time trial with MitoQ compared to placebo (each grey bar represents one individual) (**C**). * *p* < 0.05 vs placebo for paired-samples *t*-test, *n* = 19

**Fig. 4:**
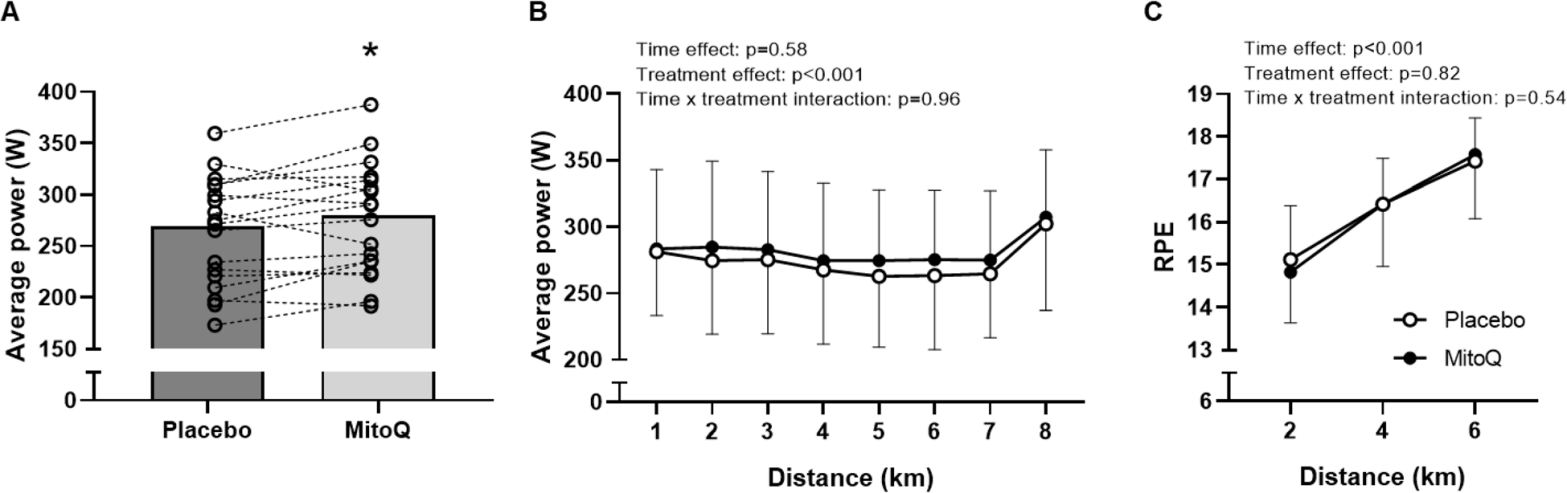
Mean power output during the time trial following 6 weeks of placebo and MitoQ supplementation (**A**), mean power output during each km of the time trial (**B**), mean RPE at 2, 4 and 6 km during the time trial (**C**). Data are presented as mean ± SD. Two-way repeated-measures ANOVA effects are given in figures and * *p* < 0.05 vs placebo for paired-samples *t*-test, *n* = 19

Time to complete the 8 km time trial was 1.3% faster following MitoQ supplementation (12.91 ± 0.94 min) compared to placebo (13.09 ± 0.95 min, *p* = 0.04, 95% CI [0.05, 2.64], *d* = 0.2, Fig. 3a-b), indicating improved performance following MitoQ supplementation. Consistent with this, 12 out the 19 participants completed the time trial faster following MitoQ supplementation compared to placebo (Fig. 3c). The mean increase in average power output during the time trial following MitoQ supplementation compared to placebo was 4.4% (placebo; 270 ± 51 W, MitoQ; 280 ± 53 W, *p* = 0.04, 95% CI [0.49, 8.22], *d* = 0.2, Fig. 4a). Moreover, a significant main effect of treatment was observed when comparing average power output during each km of the time trial (*p* < 0.001, Fig. 4b). Despite the difference in power output, there was no difference in RPE between the supplementation conditions during the time trial (Fig. 4c).

### Blood measures

**Fig. 5:**
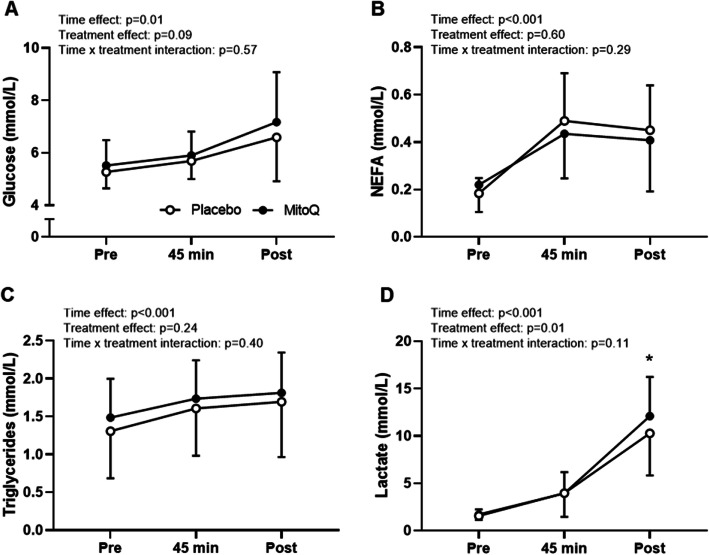
Mean plasma glucose (**A**), NEFA (**B**), triglyceride (**C**) and lactate (**D**) concentration at rest (pre), after 45 min cycling at 70% VO_2peak_ (45 min) and after the time trial (post). Data are presented as mean ± SD. Two-way repeated-measures ANOVA effects are given in figures and * *p* < 0.05 for post hoc vs placebo at the same time point, *n* = 16

A complete set of blood samples were collected from 16 participants due to technical issues with cannulas for three participants (Fig. 2). Plasma glucose, NEFA and triglyceride concentration increased during exercise (main effect for time *p* ≤ 0.01), and this was not affected by MitoQ supplementation (*p* > 0.05 for treatment and interaction effect; Fig. 5a-c). Plasma lactate concentration increased (*p* < 0.001) during exercise, and post-hoc analysis indicated that the main effect for treatment (*p* = 0.01) was a result of increased plasma lactate concentration post-exercise following MitoQ supplementation (12.09 ± 4.13 mmol·L^− 1^) compared to placebo (10.27 ± 4.45 mmol·L^− 1^, *p* = 0.04, Fig. 5d). Further analysis revealed that there was no significant difference in the slope of the linear regression lines for the association between power output during the time trial and plasma lactate concentration on completion of time trial following MitoQ vs placebo supplementation, indicating that the increased plasma lactate concentration during the MitoQ trial was the result of the increased power output.

### Lipid peroxidation

**Fig. 6:**
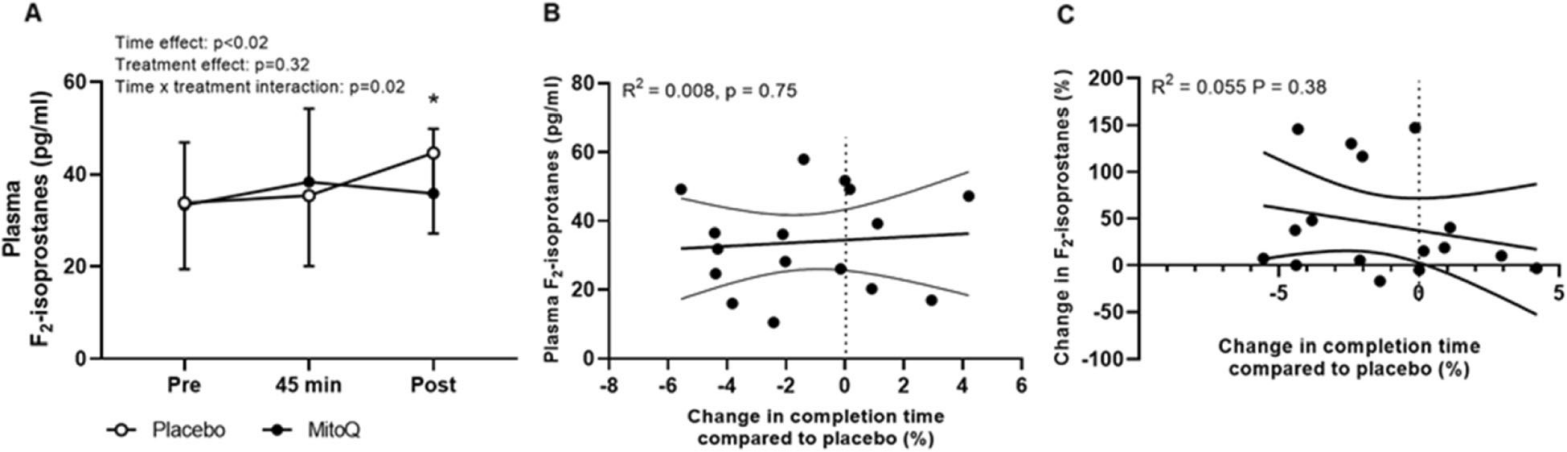
Mean plasma F_2_-isoprostane concentration at rest (pre), after 45 min cycling at 70% VO_2peak_ (45 min) and after the time trial (post), data are presented as mean ± SD (**A**), correlation between change in time to complete the time trial following MitoQ supplementation and plasma F_2_-isoprostanes at rest following placebo supplementation (**B**), and correlation between change in time to complete the time trial following MitoQ supplementation and exercise-induced changes in plasma F_2_-isoprostanes following placebo supplementation (**C**). Two-way repeated-measures ANOVA effects are given in figures and * *p* < 0.05 for post hoc vs placebo at the same time point, *n* = 16

A main effect of time and a time x treatment interaction were observed for plasma F_2_-isoprostanes (*p* < 0.05, Fig. 6a), with post hoc analysis revealing that plasma F_2_-isoprostanes were 18.31% lower on average on completion of the time trial following MitoQ (35.89 ± 14.02 pg·ml^− 1^) compared to placebo supplementation (44.68 ± 17.50 pg·ml^− 1^, *p* = 0.03, 95% CI [7.29, 29.32], *d* = 0.6, Fig. 6a). There was no association between resting plasma F_2_-isoprostanes or exercise-induced changes in plasma F_2_-isoprostanes during the performance trial and change in time to complete the time trial following MitoQ supplementation compared to placebo (*p* > 0.05, Fig. 6b and c).

## Discussion

The production of ROS by skeletal muscle during exercise is associated with the onset of muscular fatigue and a reduction in athletic performance [14]. Our study provides evidence to suggest that oral supplementation of the mitochondria-targeted antioxidant MitoQ can improve cycling time trial performance in middle-aged, recreationally trained male cyclists. The improvement in time trial performance observed in this study occurred along with an attenuation of exercise-induced increases in plasma F_2_-isoprostanes by MitoQ.

This is the first study to investigate the effect of MitoQ supplementation on cycling performance. Several studies have described improvements in cycling performance following supplementation with non-mitochondria-targeted coenzyme Q10 [31–34], which may improve performance by acting as an antioxidant within skeletal muscle mitochondria. However, accumulation of orally ingested coenzyme Q10 within mitochondria is limited by its poor bioavailability, which may explain why several others have failed to show improved performance following coenzyme Q10 supplementation [10, 35–38]. Indeed, supplementation with coenzyme Q10 is often ineffective at increasing its concentration within skeletal muscle [39–41], and several studies have shown that coenzyme Q10 supplementation does not affect exercise-induced increases in systemic markers of oxidative stress [31, 42, 43]. MitoQ has been shown to accumulate in skeletal muscle following oral administration in mice [44]. However, studies investigating the accumulation of MitoQ within skeletal muscle following oral supplementation in humans are needed.

The smallest worthwhile enhancement for cyclists competing in road time-trials has been reported to be 0.6% [45]. Therefore, the 1.3% improvement in time trial performance observed following MitoQ supplementation in this study indicates a meaningful performance improvement. However, there was significant interindividual variability in the response to MitoQ, meaning we saw a small effect of MitoQ on time trial performance (*d* = 0.2). Individual redox status seems to be an important determinant of the efficacy of antioxidant supplementation to improve performance [46]. We observed large interindividual variability in resting F_2_-isoprostanes and the redox response to exercise. However, there was no correlation between resting F_2_-isoprostanes or exercise-induced changes in F_2_-isoprostanes and change in time to complete the time trial following MitoQ supplementation compared to placebo. It should be noted that this study was not statistically powered for this outcome measure and it is important to investigate this further given that the ergogenic effects of vitamin C and N-acetylcysteine may be limited to individuals in which a specific deficiency is reversed [19]. Whether the ergogenic effects of MitoQ are limited to individuals with low levels of endogenous ubiquinone poses an interesting avenue for further investigation.

In contrast to our finding that MitoQ supplementation improves cycling performance, the mitochondria-targeted antioxidant SS-31 did not affect force production during fatiguing stimulation in isolated mouse skeletal muscle [47] or the rate of contractile force decline in intact single muscle fibres [48]. Direct comparisons between SS-31 and MitoQ are difficult given that SS-31 binds to the mitochondrial lipid cardiolipin and protects against oxidative damage whereas MitoQ protects cell membranes by acting as a chain breaking antioxidant and recycling α-tocopherol [26, 49]. However, the ergogenic effect of MitoQ may be related to the impact of MitoQ on peripheral tissues, which would not be observed in an in vitro model of muscle contraction. MitoQ has been shown to improve endothelial function in older individuals with endothelial dysfunction [50]. Whether these effects translate to healthy, physically trained individuals during exercise has not been investigated. Alternatively, the effects of mitochondria-targeted antioxidant supplementation on performance may be dependent on the mode and intensity of muscular contraction. Exercise-induced ROS production by skeletal muscle is thought to increase in an intensity-dependent manner [51], which may explain why we saw an improvement in performance during high intensity exercise following MitoQ supplementation while the physiological response to cycling at 70% VO_2peak_ was unchanged. On the other hand, the higher plasma lactate concentration coupled with improved time trial performance with MitoQ suggests that MitoQ may improve performance by allowing for increased use of anaerobic metabolism during high intensity exercise, or by improving tolerance to lower pH in skeletal muscle. Future studies should aim to clarify whether the positive effects of MitoQ on performance and markers of exercise-induced oxidative stress are limited to exercise at a high intensity.

A major finding of the current study is that MitoQ attenuated the increase in plasma F_2_-isoprostanes during the time trial, which may indicate a protective effect of MitoQ against exercise-induced lipid peroxidation within mitochondria. Mitochondrial lipids are essential for maintaining the integrity of mitochondrial membranes and proper function of mitochondria; however, they are prone to lipid peroxidation by free radicals [52]. Therefore, by attenuating exercise-induced lipid peroxidation in mitochondria, MitoQ may preserve the structure and dynamics of mitochondrial membranes and the efficiency through which mitochondria can supply ATP for muscular contraction. However, plasma F_2_-isoprostanes are a systemic marker of lipid peroxidation, meaning the post-exercise increase in plasma F_2_-isoprostane levels may also be derived from non-mitochondrial sources. Similar to previous studies [29, 53], we saw no effect of MitoQ on pre-exercise markers of oxidative stress. However, given that the participants were healthy and exercise-trained, it seems likely that any changes in resting F_2_-isoprostanes would have been difficult to detect [54]. It appears that MitoQ may be most effective in lowering oxidative stress in situations where mitochondria are under stress [55].

Our study has limitations that should be acknowledged. Participants were all recreationally trained men and caution should be taken when extrapolating these results to elite athletes and females. Furthermore, the inclusion of a performance test at the start of each supplementation phase would have enabled us to ensure that any lasting effects of MitoQ supplementation were not carried over. A 6-week washout period has been shown to reverse the effects of MitoQ supplementation on mitochondrial H_2_O_2_ levels [53]. However, we cannot be sure that MitoQ supplementation did not result in adaptations that may have lasted beyond the washout period. Participants supplemented for 28 days while maintaining their habitual training, which means that we cannot determine whether the improved cycling performance reflects an ergogenic effect of MitoQ on exercise performance alone or an interaction between MitoQ and training. The contents of the MitoQ and placebo tablets used in this study was not verified by an independent agent, which means that a risk of contamination cannot be excluded. Finally, we did not quantify the level of MitoQ in skeletal muscle biopsies and cannot confirm that MitoQ was present within the skeletal muscle of the participants during the performance trial.

## Conclusions

Taken together, our work provides evidence that MitoQ can attenuate exercise-induced increases in lipid peroxidation and improve cycling time trial performance in middle-aged, recreationally trained men. It appears that MitoQ may improve performance above the aerobic threshold by allowing for increased use of anaerobic metabolism, or by improving tolerance to lower pH in muscle. Further research is required to determine the mechanisms responsible for the ergogenic effect of MitoQ, understand the important factors that determine an individual’s response to MitoQ supplementation, and identify optimal dosing strategies.

## Data Availability

Data and publication materials are available from the corresponding author on reasonable request.

## Abbrieviations

H_2_O_2_: Hydrogen peroxide
NEFA: Non-esterified fatty acids
O_2_·^−^: Superoxide
RER: Respiratory exchange ratio
ROS: Reactive oxygen species
RPE: Rating of perceived exertion
